# Persistent, neuropathic-like trigeminal pain after dental implant loading

**DOI:** 10.4317/jced.59248

**Published:** 2022-02-01

**Authors:** Ndiaye Amadou-Diaw, Adeline Braud, Yves Boucher

**Affiliations:** 1 MSc. UFR d’Odontologie, Université de Paris; 2 DDS, PhD. Laboratoire de Neurobiologie Orofaciale (EA7543), UFR Odontologie, Université de Paris & Service Odontologie, Hôpital Rothschild, APHP, Paris, France; 3 DDS, PhD. Laboratoire de Neurobiologie Orofaciale (EA7543), UFR Odontologie, Université de Paris & Service Odontologie, Hôpital Pitié Salpêtrière, APHP, Paris, France

## Abstract

**Background:**

Painful post-traumatic trigeminal neuropathy (PTTN) is a known complication of dental implant therapy. Besides cases resulting of nerve damage during surgery or implant placement, some patients report delayed neuropathic-like symptoms only after implant loading i.e. crown placement. The present retrospective study aimed at describing the clinical features of pain experienced by these patients.

**Material and Methods:**

The cohort of patients consulting for chronic orofacial pain at the Groupe Hospitalier Pitié-Salpêtrière (Paris, France) between 2015 and 2020 (ABCD study, IRB # TPS 1106180), was screened for patients with history of dental implant placement and persistent pain. Patients with no pain after surgery for 6 months and pain resulting from the loading of the implant, were included.

**Results:**

Among 675 files of patients screened, 5 fulfilled inclusion criteria. All patients were women, mean age 62.4 ± 9.8 y.o, and reported trigeminal neuropathic-like persistent pain. Intensity of pain was described as moderate to severe, with pin and needles, burning and tingling, and electric shocks as main symptoms.

**Conclusions:**

These results suggest that implant loading can trigger trigeminal neuropathy, in a previously sensitized nerve. Putative neurophysiological basis of the phenomenon is discussed.

** Key words:**Neuropathic pain, trigeminal nerve, lesion, dental implant, implant loading, allodynia.

## Introduction

Dental Implant therapy (DIT) has become over the years a well-defined and accepted therapy with increasing indications. The success rate of DIT is overall high and the failures are mainly due to periimplantitis and treatment planning errors (1,2). Post-operative surgical pain usually resolve in a few days or weeks (3) but persistent pain can also occur, mainly as a result of nerve injury (4-6). Indeed DIT is suggested to be the second cause of trigeminal nerve injury and sensory disturbances, after third molar extraction (7-10). Its incidence ranges from 0.8% to 33% (6,9).

Painful Post Traumatic Trigeminal Neuropathy (PTTN) has been defined in the ICHD3, as persistent pain occurring in a delay of 3-6 months in the area of a documented trigeminal nerve lesion (11,12). This type of neuropathic pain (NP) has a variable clinical phenotype, either in intensity, temporal pattern or quality, with patients experiencing mild to severe continuous or intermittent pain, reported frequently as burning, pricking or tingling sensations, electric shocks, paresthesia, and/or mechanical allodynia (7,12,13). The individual and societal impact of PTTN is nowadays recognized with impaired psychosocial functioning, affective behavior, food ingestion and communication, associated with decreased quality of life (6,9,14).

PTTN after DIT is usually caused by trigeminal nerve damage during implant surgery either by direct nerve lesion caused by the drill or the implant or by indirect damage. The latter may have different origins for example compression of the nerve by bone debris or edema , thermal injury caused by local heat during implant drilling (2,15,16), leading in fine to release of neuro-inflammatory mediators.

Besides these cases in which persistent pain can be attributed to the nerve damage caused by implant surgery, we noticed in our secondary orofacial pain consultation that several patients reported onset of trigeminal neuropathic-like pain only after implant loading, i.e. after the placement of the crown or bridge; the pain thus could not be attributable to nerve damage. This intriguing observations prompted us to document the phenomenon. A retrospective cohort study was therefore undertaken in order to provide a clinical description of these patients.

## Material and Methods

This cross sectional observational retrospective cohort study was approved by an IRB (CPP, protocol ABCD, INDS: TPS 1106180), registered in the national data protection agency (CNIL), and followed the STROBE recommendations for observational studies. It complied with the ethical principles of the Helsinki declaration and Good Clinical Practice. Anonymity of participants was respected throughout the course of the study.

-Patient selection 

The COFP consultation of the dental service of the Pitié-Salpêtrière Hospital (GHPS) is a clinical setting for either inpatients or outpatients, mainly referred by dentists, physicians (GP or specialists) for complex pain. Clinical records from 2015 to 2020 were screened between September 2019 and June 2020 according to the following inclusion and exclusion criteria.

Inclusion criteria.

• history of dental implant placement 

• absence of pain before implant placement 

• pain in the area of implants 

• absence of persistent pain in a 6-month period after implant placement, except in the 3 weeks following surgery

Exclusion criteria.

• Patients under 18 y.o.

• Patients with impaired communication 

• Pain with pain before implantation and/or loading

• Pain occurring within 4 weeks after dental implant loading

• Patients suffering from other chronic painful oral disease, unless well identified such as DTM 

• Pain arising from another region than the trigeminal nerve.

• Pain arising from a different branch of the trigeminal nerve than concerned by the implant 

• Periimplantitis, assesssed clinically and radiographically 

• Interference of the implant with the nerve trajectory suggestive of a nerve lesion 

-Data extraction 

The data collection included demographic data (sex, age); medical status, dental and loco-regional history; medications; clinical features of pain including intensity assessed on a 0-10 numerical scale (NS), quality, temporal pattern, location, delay of apparition and time course; associated autonomic signs; aggravating and relieving factors; DN4 questionnaire as used in a previous study for chronic orofacial pain (17).

## Results

-Sample 

Among the 675 patients attending the consultation during the period of the study, 5 fulfilled inclusion criteria and constituted the final sample. They were all women, aging from 53 to 74 years with medical characteristics and pain phenotypes reported in Table 1 and  Table 2.

**Table 1:**
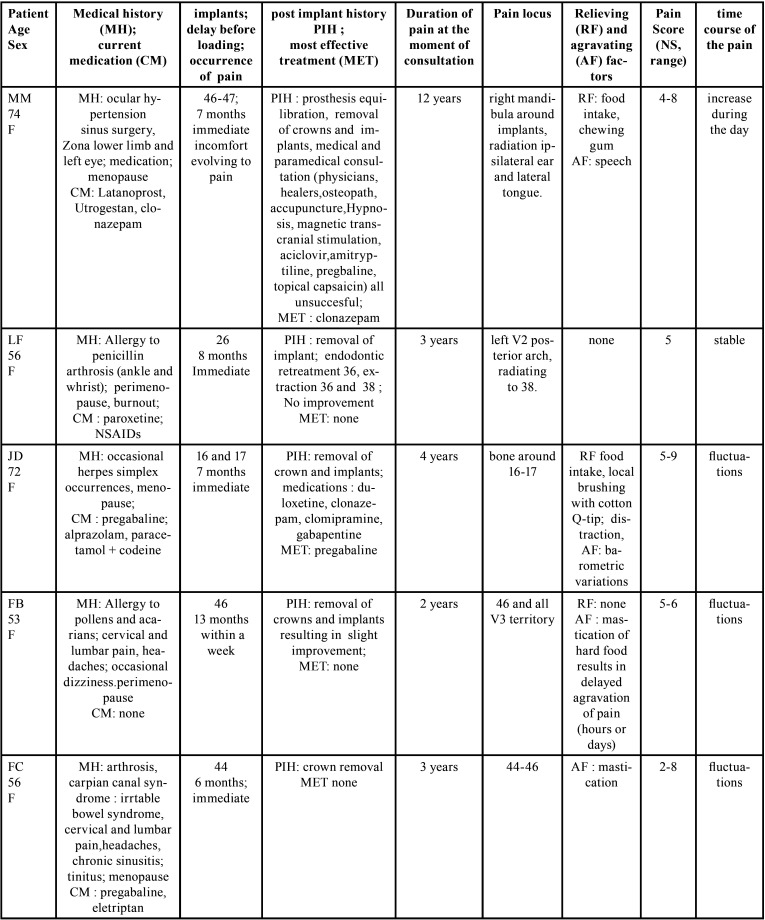
Characteritics of the patients suffering from neuropathic-like pain after dental implant loading.

**Table 2:**
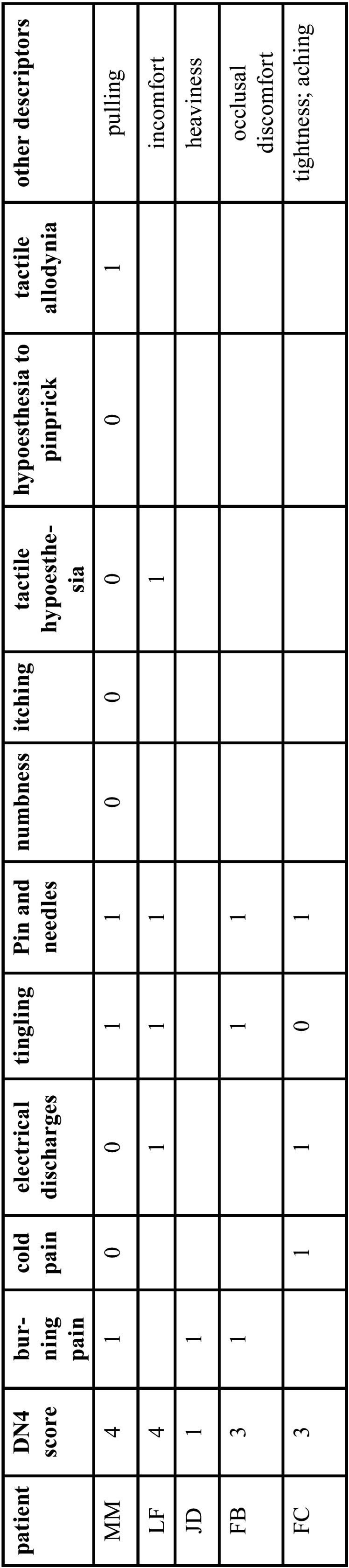
DN4 scores of the pain experienced by patients suffering from neuropathic-like pain after dental implant loading.

-Onset of the pain

no patient reported a painful surgery and a six-month period minimum was respected between the implant loading, with no pain during this period (inclusion criteria). All patients experienced oral discomfort a few days after implant loading, and complained to their practitioner. Prosthetic elements were removed in all cases without long term improvement. Implants were removed in 4 cases resulting in a reduction in painful sensations for 2 patients. In order to relieve pain, the patients consulted several specialists such as GP, stomatologists, healers, osteopath, acupuncturist, changed dentist, were referred to a pain center were specific prescriptions were given.

-Pain phenotype 

All patients reported continuous pain, either stable (1 patient) increasing during the day (1 patient) or with fluctuations with no specific temporal pattern (3 patients). Intensity ranged from 2 to 9 on the 0-1 NS, oral function (speech and mastication) exacerbated the pain in 2 patients when food intake (meal) relieved the pain in two patients. One had no aggravating/exacerbating factors). The most described sensations were pin and needles (4/5) > burning pain and tingling (3/5) > electric shocks (2/5)>cold pain and mechanical sensation not captured by DN4 described as incomfort, heaviness, tightness, pulling

-Localization

All patients reported pain in the area of the implantation site, at the maxilla or mandible. In two patients the pain area was circumscribed to this site when it radiated in the ipsilateral trigeminal branch in three patients and also in the opposite arch in one patient.

-Treatments.

Classical treatments for NP were attempted (clonazepam, pregabaline, gabapentine, aciclovir, hypnosis, or transcranial magnetic stimulation) but none were satisfactorily although the pain was attenuated in 2 patients.

## Discussion

In this retrospective study, we identified a subgroup of patients with persistent orofacial pain after dental implant therapy evoking PTTN although not attributed to the surgical procedure per se, but by implant loading, i.e. after the fitting of the implant-supported prosthetic crown. A similar case has previously been mentioned in the literature (18).

-Clinical considerations

According to the symptoms and clinical behavior, the patients were diagnosed as NP in line with IASP recommendations (19). Although there is no yet unique semiology for NP, the patients reported neuropathic-like symptoms i.e. moderate to intense persistent spontaneous pain, allodynia, with pin and needles, burning, tingling as main descriptors (12,20). In addition pain was not relieved by classical analgesics which is a key feature of NP, and poorly responded to non-classical analgesics which is also frequent in orofacial NP (21). Except for criteria C2, (Table 3) i.e. the time window for pain appearance, the patients fulfilled the ICHD criteria of PTTN (11). They felt a persistent pain in the territory of the trigeminal branch with a history of a traumatic event, i.e. extraction and/or implant. It is now acknowledged that even minor surgeries elicit nerve lesions, despite no major nerve trunk damage and this seems especially true in the densely innervated trigeminal region. For example taking account of dental nerve deafferentations occurring after root canal treatments and extractions resulted in the conceptual shift from Atypical Odontalgia to PPTN in orofacial pain taxonomy (13). However in the present study, the observed symptoms cannot be attributed to the surgical procedure alone since 1) no patient reported episode of pain during surgery evocative of a nerve lesion (2) and 2) no pain occurred in the 6 months period which defines PTTN; it can be added that in most cases of PTTN the pain occurs before (12). Thus, other mechanisms must be proposed.

**Table 3:**
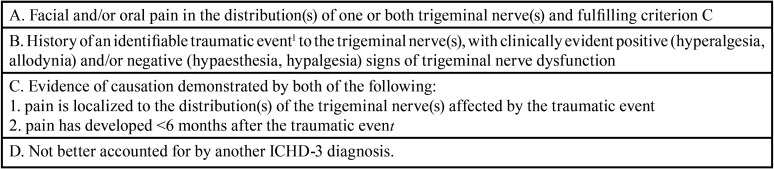
Painful post traumatic trigeminal neuropathy criteria according to ICHD3.

-Pathophysiological hypotheses

A simple explanation is difficult to find, first because there was no identical semiology, either for intensity, pattern or quality; second, because further interventions were sometimes performed in order to relieve the pain (example JD extraction 38). It is therefore possible that these interventions worsened the initial symptomatology. It is also known that neuropathic changes elicit a diversity of sensations reflecting the diversity of mechanisms and types of nerve fibers involved (20). Keeping these limitations in mind, several hypotheses can be examined.

The pathophysiology of PTTN triggered by nerve lesions is complex and involves peripheral and central mechanisms (12). Among them, alteration of neurovascular units, resulting in long term genetic and epigenetic changes phenotypic changes in the damaged tissues, trigeminal ganglion and sensory trigeminal complex, through numerous bioactive molecules including proteins, lipids, chemokines, mRNA, LTRNA (22-25) have been described.

An interesting observation is provided by Tashima *et al*. (26) who showed that Aß nerve fibers activation through optogenetics after a nerve lesion can lead to neuropathic pain behavior. Thus a non-painful mechanical stimulation usually mediated by Aß fibers can lead to a painful sensation. In physiological conditions, mechanical sensations are conveyed by large myelinated A-fiber and interoceptive (homeostatic) sensations (including pain, itch, temperature, chemical sensing, pleasant touch etc.) by small diameter afferents. These two phylogenetically different systems, intermingled through evolution, communicating with each other and contributing to global body perception (27), have segregated pathways at the spinal/trigeminal level, responsible for the specificity of the sensations. They can be functionally reconfigured in pathological states such as nerve lesions, through activation/inhibition of interneurons and glial cells, with specificities according to the nature of the injury (28,29). Changes in the spinal/trigeminal system, involving for example GABA and PKCγ interneurons and glial cells (30-35), have been documented. This changes can explain why a mechanical sensation can lead to persistent pain (36).

If neuropathic changes in primary afferents and spinal/trigeminal are well documented, other mechanisms involving suprasegmental neuronal networks might also occur. It is for example known that tooth removal and its replacement by implant both lead to peripheral nerve degenerative/regenerative pathways as well as somato-sensory cortex reorganization (37). Alteration of ascending and descending pain controls play an important role in persistent NP (28,38). In this regard, it is interesting to note that two patients in this study were relieved during meals, suggesting a central mechanism (39).

-Priming 

Since the symptoms did not appear immediately, the concept of priming can be evoked. As previously proposed in other conditions, the inflammatory sensitization of primary afferents might favor the occurrence of NP in case of a later traumatic event (40). In the cases reported here, the release of inflammatory mediators elicited by implant surgery (15,41,42) i.e. cytokines, endothelins, growth factors, prostanoids, hypoxic factors etc. might have sensitized nociceptors at the subclinical level. Of interest is the chemokine CCL2, released by damaged neurons in neuropathic pain models. It elicits long term changes in primary afferents and plays a major role in neuropathic pain and allodynia development (43,44). Interestingly, CCL2 is also released, with different characteristics, in nociceptive or surgical conditions (45,46).Then, the surgery and associated neuronal damage might have modified at a subclinical level the trigeminal afferents, which revealed clinically after the mechanical stimulation caused by implant loading. Such observations provide support for the clinical cases presented here, i.e. turning a mechanical non-painful stimulation into persistent pain. Other mechanisms can be evoked, including the involvement of the mechanoreceptor Piezo2 which is expressed in the majority of Aδ small-diameter myelinated nociceptors, including those that innervate the bone marrow and is sensitized by inflammatory mediators (47).

-Limitations.

The retrospective study design limits the collection of clinical information. Main data are declarative patient-based data.

## Conclusions

This study supports the hypothesis that implant loading may be responsible for the development of neuropathic pain. A two-step mechanism can be evoked with a sensitization of primary sensory afferents by neuro-inflammatory mechanisms, secondary activated by mechanical afferents after implant loading.
